# LRRK2 kinase activity regulates lysosomal glucocerebrosidase in neurons derived from Parkinson’s disease patients

**DOI:** 10.1038/s41467-019-13413-w

**Published:** 2019-12-05

**Authors:** Daniel Ysselstein, Maria Nguyen, Tiffany J. Young, Alex Severino, Michael Schwake, Kalpana Merchant, Dimitri Krainc

**Affiliations:** Department of Neurology, Northwestern University Feinberg School of Medicine, 303 E Chicago Avenue, Chicago, IL 60611 USA

**Keywords:** Parkinson's disease, Molecular neuroscience

## Abstract

Mutations in *LRRK2* and *GBA1* are common genetic risk factors for Parkinson’s disease (PD) and major efforts are underway to develop new therapeutics that target LRRK2 or glucocerebrosidase (GCase). Here we describe a mechanistic and therapeutic convergence of LRRK2 and GCase in neurons derived from patients with PD. We find that GCase activity was reduced in dopaminergic (DA) neurons derived from PD patients with *LRRK2* mutations. Inhibition of LRRK2 kinase activity results in increased GCase activity in DA neurons with either *LRRK2* or *GBA1* mutations. This increase is sufficient to partially rescue accumulation of oxidized dopamine and alpha-synuclein in PD patient neurons. We have identified the LRRK2 substrate Rab10 as a key mediator of LRRK2 regulation of GCase activity. Together, these results suggest an important role of mutant LRRK2 as a negative regulator of lysosomal GCase activity.

## Abstract (web summary)

Mutations in *LRRK2* and *GBA1*, which encodes glucocerebrosidase (GCase), are associated with Parkinson’s disease. Here the authors show that LRRK2 is a negative regulator of lysosomal GCase activity, using dopaminergic neurons derived from iPSCs from PD patients with *LRRK2* mutations.

## Introduction

Parkinson’s disease (PD) is the most common neurodegenerative movement disorder that is pathologically characterized with early loss of dopaminergic neurons in the substantia nigra pars compacta (SNc)^1^. The identification of a growing number of genetic forms of the disease^2,3^ has provided valuable insights into the cellular pathways that are altered in the disease process and can aid the search for new therapeutic strategies for PD.

Mutations in the gene encoding leucine rich repeat kinase 2 (*LRRK2*) represent one of the most common genetic causes of PD. LRRK2 is a 286 kDa protein with several functional domains including a kinase domain^4^. To date, over 50 different mutations in the *LRRK2* gene have been reported^5^, with the G2019S point mutation being the most common pathogenic mutation^5–8^. Pathogenic mutations increase LRRK2 kinase activity and hence have been classified as gain-of-function mutations^9,10^. Recently, increased LRRK2 kinase activity was observed in idiopathic PD and in neurons exposed to mitochondrial toxins, highlighting the possibility of a broader role of LRRK2 kinase activity in PD pathogenesis^11^. Despite the significance of LRRK2 in PD, its physiologic function or pathogenic mechanism underlying PD is not fully elucidated. Increasing evidence suggests a role for LRRK2 in synaptic function^12^ and endo-lysosomal trafficking^13^, although LRRK2 has also been implicated in cellular proliferation^14^, inflammation^15^, and cytoskeleton dynamics^16^. Unfortunately, the uncertainty in the precise role of LRRK2 is not resolved by *LRRK2* transgenic or knock-in mouse models due to the lack of a common and consistent phenotype across mouse lines and the inability to recapitulate degeneration of nigral dopaminergic (DA)  neurons or synuclein pathology observed in patients with PD^17,18^. We have recently shown that human DA neurons differentiated from induced pluripotent stem cells (iPSCs) exhibit pathological phenotypes such as accumulation of oxidized dopamine products  and neuromelanin that are also observed in PD autopsied brain tissue but not seen in mouse models^19^.

The most common risk factor for PD is mutations in the gene *GBA1*, which encodes for glucocerebrosidase 1 (GCase). GCase is a lysosomal hydrolase that catalyzes the hydrolysis of glucosylceramide to glucose and ceramide. Homozygous mutations in GCase that lead to a loss of function result in a lysosomal storage disorder called Gaucher’s disease. Heterozygous point mutations represent a major genetic risk factor for PD^20^ with 7–10% of PD patients harboring a mutation in *GBA1*^21^. Additionally, a growing number of patients have been reported to have concurrent *LRRK2* G2019S mutation with either *GBA1* L444P^22^ or E326K mutations^23^. These patients developed PD symptoms at an earlier age compared to carriers of only *LRRK2* or *GBA1* mutations^22–24^. Based on these observations, we hypothesized that *GBA1* and *LRRK2* mutations may contribute to PD pathogenesis through a common biological pathway.

To test this hypothesis, we examined GCase activity in DA neurons derived from PD patients and found that *LRRK2* mutations result in reduced lysosomal GCase activity. Inhibition of LRRK2 kinase activity significantly restored GCase activity in neurons that carry mutations in *LRRK2* or *GBA1*, leading to rescue of PD-associated phenotypes. Our results highlight the potential for LRRK2 inhibition in idiopathic and *GBA1*-PD and for GCase activation in *LRRK2* patients. These findings could have significant therapeutic implications for these patient populations as therapeutic compounds targeting either LRRK2 or GCase are currently in clinical trials.

## Results

### GCase activity is reduced in DA neurons with *LRRK2* mutations

**Fig. 1:**
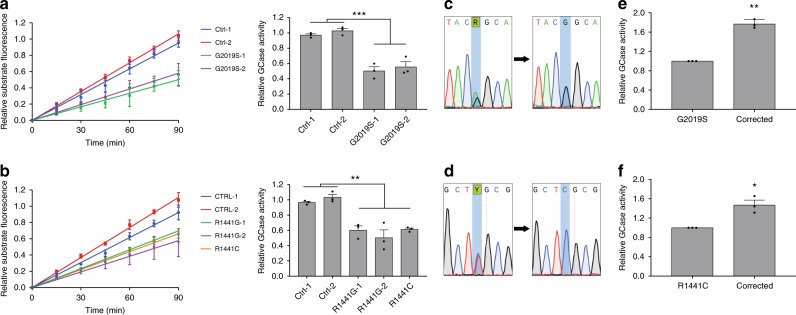
GCase activity is reduced in DA neurons with *LRRK2* mutations. Live-cell measurement of fluorescent unquenching resulting from hydrolysis of the artificial GCase substrate PFB-FDGlu by lysosomal GCase in LRRK2 G2019S (**a** left panel) and R1441G/C (**b** left panel) DA neurons relative to healthy controls over 90 min. GCase activity was determined by analyzing the relative slope of these measurements (**a**, **b** right panel). Sanger sequencing results from *LRRK2* G2019S **c** and R1441C **d** lines  and subsequent isogenic controls generated using CRISPR/Cas9. Relative lysosomal GCase activity in DA neurons with LRRK2 G2019S **e** and R1441C **f** mutations compared to isogenic corrected controls. The data are presented as the mean ± SEM, *n* = 3; *p < 0.05, **p < 0.01, ***p < 0.001, using one-way ANOVA followed by Tukey’s multiple comparison *post hoc test* **a**, **b**, or paired two-tailed *t*-test **e**, **f**. Source data are provided as a Source Data file.

Since patients that carry concurrent *LRRK2* and *GBA1* mutations develop PD symptoms at an earlier age compared to carriers of single mutations, we first examined the potential role of GCase in LRRK2-mediated disease pathogenesis. To this end, fibroblasts were obtained from PD patients carrying *LRRK2* G2019S, R1441C, and R1441G mutations along with healthy controls. Fibroblasts were reprogrammed to iPSCs and then differentiated into dopaminergic neurons^25^ that were maintained in long-term cultures and analyzed at day 90 post differentiation. We have previously found that these neurons faithfully recapitulate PD disease phenotypes^19,26^. Lysosomal GCase activity in live cells was measured using the fluorescent quenched substrate PFB-FDGlu that enables real-time analysis of lysosome-specific GCase activity^27^, unlike traditional approaches which measure activity in lysed cells. Using this approach, we examined the effects of LRRK2 G2019S mutations on GCase activity in mutant versus control DA neurons and observed a significant reduction in GCase activity in two independent *LRRK2* G2019S iPSC neuronal lines relative to two distinct healthy controls (Fig. 1a). To examine whether this effect was specific to the G2019S mutation, we examined GCase activity in neurons containing LRRK2 R1441G and R1441C mutations. These mutations also displayed a significant reduction in GCase activity relative to healthy controls (Fig. 1b). In order to generate additional relevant controls, we used CRISPR/Cas9 to correct the point mutations in *LRRK2* G2019S and R1441C iPSCs (Fig. 1c, d). Neurons differentiated from these isogenic lines displayed very similar LRRK2 expression levels (Supplementary Fig. 3b) and showed a significant recovery in GCase activity for both G2019S (Fig. 1c, e) and R1441C mutations (Fig. 1d, f). Collectively, these results indicate that lysosomal GCase activity is reduced in human DA neurons derived from iPSCs with *LRRK2* mutations.

### Inhibition of LRRK2 increases GCase activity in DA neurons

**Fig. 2:**
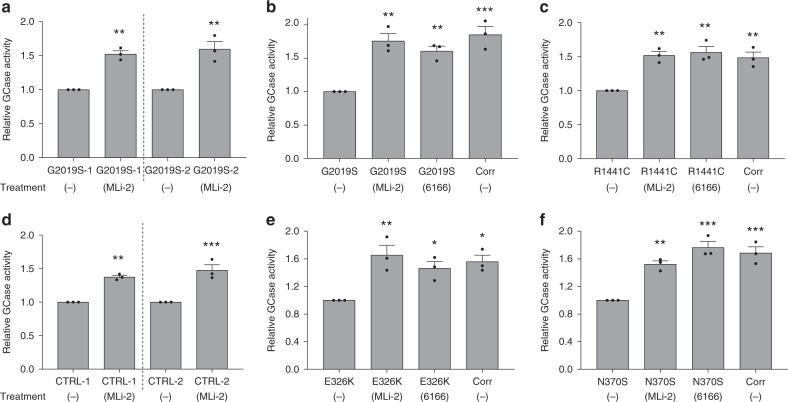
Inhibition of LRRK2 kinase activity increases GCase activity in DA neurons from PD patients. Examination of lysosomal GCase activity in LRRK2 G2019S DA neurons treated with the LRRK2 kinase inhibitor, MLi-2 (600 nM) **a**. Comparison of GCase activity in LRRK2 G2019S **b** and R1441C **c** DA neurons relative to isogenic controls and neurons treated with MLi-2 or a direct GCase modulating compound, 6166 (3 µM). GCase activity measured in DA neurons from two independent healthy controls treated with MLi-2 **d**. Comparison of GCase activity in GBA1 E326K **e** or N370S **f** DA neurons relative to isogenic controls and neurons treated with MLi-2 or 6166. The data are presented as the mean ± SEM, *n* = 3; *p < 0.05, **p < 0.01, ***p < 0.001 relative to untreated, one-way ANOVA followed by Tukey’s multiple comparison *post hoc test*. Source data are provided as a Source Data file.

Both G2019S and R1441C mutations increase the kinase activity of LRRK2^9,10^. To examine whether the kinase function plays a role in modulating GCase activity in LRRK2 mutant DA neurons, we treated LRRK2 G2019S neurons with a potent and well-characterized LRRK2 kinase inhibitor, MLi-2^28^. Interestingly, MLi-2 treatment increased GCase activity in two separate DA neuronal cell lines with each *LRRK2* mutation (Fig. 2a.). We compared the efficacy of MLi-2 with a modulator of GCase, 6166, developed in our lab^29^, as well as the mutation corrected LRRK2 G2019S and R1441C isogenic control lines. Inhibition of LRRK2 kinase function restored GCase activity to levels observed in respective isogenic control neurons, as well as to levels induced by the GCase modulator (Fig. 2b, c). Next, we tested the specificity of LRRK2 kinase inhibition effects on GCase activity in DA neurons by testing the effects of MLi-2 on neurons with wild-type (WT) LRRK2 or mutant GCase. Surprisingly, treatment of healthy control DA neurons with the LRRK2 inhibitor also led to a significant increase in GCase activity in these cells (Fig. 2d). We then examined the effect of LRRK2 kinase inhibition on neurons carrying common heterozygous mutations in the *GBA1* gene, E326K or N370S^30^. To evaluate the significance of any effect on GCase activity, isogenic control lines for *GBA1* E326K and N370S were generated (Supplementary Fig. 1c, d) and simultaneously differentiated into DA neurons (Supplementary Fig. 2c, d). As expected, correction of *GBA1* E326K and N370S mutations significantly increased GCase activity by 54% and 68% respectively (Fig. 2e, f). Interestingly, treatment of these GBA1 mutant DA neurons with MLi-2 increased GCase activity to levels comparable to those observed in isogenic control neurons (Fig. 2e, f). A similar level of increase was induced by the GCase modulator, 6166 (Fig. 2e, f). Importantly, LRRK2 expression level was unchanged in these neurons (Supplementary Fig. 3b). Together, these results highlight an important role of LRRK2 in regulation of GCase activity in human DA neurons, independent of mutation status or disease state.

### LRRK2 inhibition rescues PD-related phenotypes in DA neurons

**Fig. 3:**
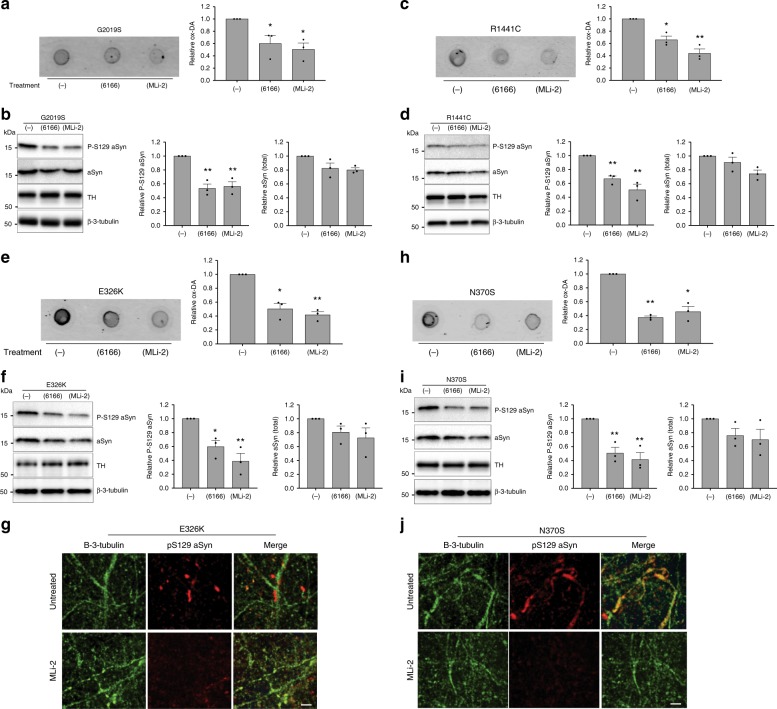
LRRK2 kinase inhibitors rescue PD-related pathophysiologic phenotypes in LRRK2 and GBA1 mutant neurons. Measurement of insoluble oxidized dopamine by near-IR fluorescence from LRRK2 G2019S. **a** and R1441C **c** mutant DA neurons treated with 6166 or MLi-2. Treated cultures were also subjected to western blot analysis of phospho-S129 aSyn (P-S129), total aSyn, and tyrosine hydrolase (TH) with β-3-tubulin used as a loading control **b**, **d**. Measurement of relative levels of insoluble oxidized dopamine by near-IR fluorescence from DA neurons containing *GBA1* E326K **e** or N370S **h** mutations. Treated cultures were also subjected to western blot analysis of P-S129, total aSyn, and TH with β-3-tubulin used as a loading control **f**, **i**. Representative images from additional DA neurons containing GBA1 E326K **g** or N370S **j** mutations treated with MLi-2 and stained with antibodies targeted to P-S129 and β-3-tubulin, scale bars, 50 µm. The data are presented as the mean ± SEM, *n* = 3; *p < 0.05, **p < 0.01 relative to untreated, one-way ANOVA followed by Tukey’s multiple comparison *post hoc test*. Source data are provided as a Source Data file.

We have previously shown that iPSC-derived DA neurons from PD patients accumulate two pathophysiologic markers; dopamine oxidation products^19^ and phospho-Ser129-alpha synuclein. Interestingly, treatment with a GCase modulator was found to mitigate both phenotypes. To test whether inhibition of LRRK2 kinase activity could also reduce these pathophysiologic markers, we compared the effects of MLi-2 and 6166 in DA neurons from patients with *LRRK2* or *GBA1* mutations. When compared to vehicle-treated DA neurons, MLi-2 or 6166 treatment significantly reduced accumulation of dopamine oxidation products (Fig. 3a, c), as well as phospho-Ser129 alpha synuclein (Fig. 3b, d) in DA neurons with a LRRK2 G2019S or R1441C mutation. Moreover, neurons that carry a *GBA1* E326K or N370S mutation also showed a significant reduction in accumulation of dopamine oxidation and phospho-S129 alpha synuclein measured by western blot and immunocytochemistry in response to MLi-2 and 6166 (Fig. 3e–j). Together, these results indicate that LRRK2 kinase inhibitors or GCase modulators have the potential to protect DA neurons with *LRRK2* or *GBA1* mutations.

### GCase activity is negatively regulated by LRRK2 activity

**Fig. 4:**
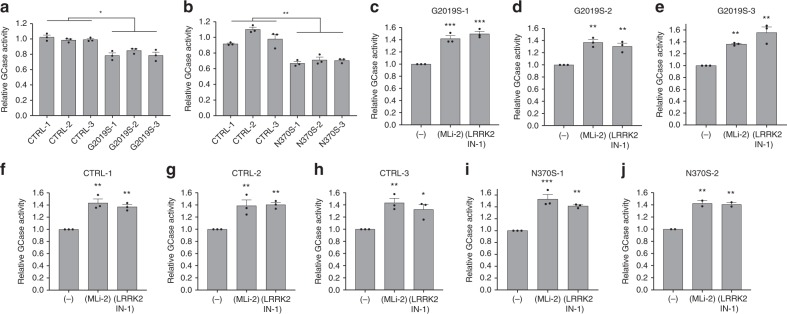
GCase activity is negatively regulated by LRRK2 kinase activity in patient-derived fibroblasts. Comparison of live-cell lysosomal GCase activity in fibroblasts derived from healthy control individuals, as well as patients with *LRRK2* G2019S **a** and *GBA1* N370S **b** mutations. GCase activity in patient-derived fibroblasts with *LRRK2* G2019S mutations treated with either the LRRK2 inhibitors MLi-2 (600 nM) or LRRK2-IN-1 (2 µM) **c**–**e**. GCase activity measured in additional cultures derived from healthy controls **f**–**h** and from individuals with *GBA1* N370S mutations **i**, **j** treated with either MLi-2 or LRRK2-IN-1. The data are presented as the mean ± SEM, *n* = 3 (*n* = 2 for N370S-2); *p < 0.05, **p < 0.01, ***p < 0.001 relative to healthy controls **a**, **b** or untreated **c**–**j**, with one-way ANOVA followed by Tukey’s multiple comparison *post hoc test*. Source data are provided as a Source Data file.

The results described above reveal that LRRK2 kinase activity negatively regulates GCase activity in DA neurons. To examine whether the effect of LRRK2 on GCase activity is specific to DA neurons we examined GCase activity in fibroblasts from PD patients with and without *LRRK2* mutations. As a reference, we also measured GCase activity in fibroblasts from individuals with the *GBA1* N370S mutation. Surprisingly, we observed a small (20%), but statistically significant reduction in GCase activity in three separate lines with G2019S mutation relative to WT controls (Fig. 4a). This reduction was not as dramatic as the effect of the *GBA1* N370S mutation (Fig. 4b). However, the data indicate that the effect of LRRK2 mutations on GCase activity is not specific to iPS-derived DA neurons. To determine the role of LRRK2 kinase function in the regulation of GCase activity in the fibroblasts, we examined the effect of two distinct LRRK2 kinase inhibitors, MLi-2 and LRRK2-IN-1. Both LRRK2 kinase inhibitors consistently increased lysosomal GCase activity in the fibroblasts from PD patients with *LRRK2* G2019S mutation (Fig. 4c-e), healthy controls (Fig. 4f-h) or PD patients with *GBA1* N370S mutation (Fig. 4i, j). These results suggest that LRRK2-mediated decrease of GCase activity may involve mechanisms that are not only specific to DA neurons.

### LRRK2 regulation of GCase activity is mediated by Rab10

**Fig. 5:**
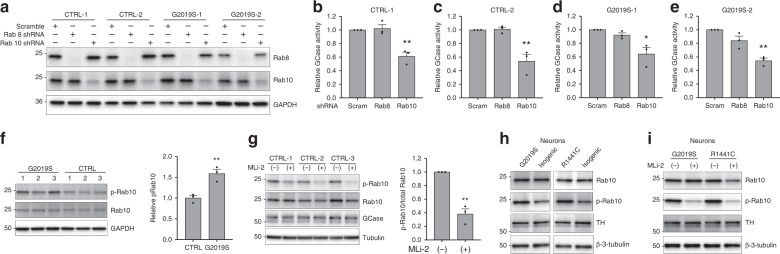
Rab10 is a mediator of GCase activity by LRRK2 in fibroblasts and DA neurons. Western blot analysis of fibroblasts, from healthy controls or from patients with the *LRRK2* G2019S mutation treated with lentivirus encoding Rab8 and Rab10 shRNA, probed for Rab8, Rab10, and GAPDH (loading control) **a**. Examination of relative lysosomal GCase activity in fibroblasts upon Rab8 and Rab10 knock-down **b**–**e**. Western blot analysis of fibroblasts from 3 patients with *LRRK2* G2019S and from 3 healthy controls were probed for phospho-Rab10 (p-Rab10), Rab10, and GAPDH (loading control). The data is presented as the average p-Rab10 signal for the 3 G2019S samples relative to the 3 controls **f**. Western blot analysis of fibroblasts from 3 controls treated with MLi-2 were probed for phospho-Rab10 (p-Rab10), Rab10, GCase, and tubulin (loading control). Data is presented as the ratio of p-Rab10 to total Rab10 for the treated relative to untreated cells **g**. Representative western blots of lysates from LRRK2 G2019S and R1441C DA neurons relative to the corresponding isogenic controls **h** or relative to MLi-2 treated neurons **i** were probed for p-Rab10, Rab10 and β-3-tubulin (loading control). The data are presented as the mean ± SEM, *n* = 3; *p < 0.05, **p < 0.01, using one-way ANOVA followed by Tukey’s multiple comparison *post hoc test* **b**–**e**, or paired two-tailed *t*-test **f**, **g**. Source da*t*a are provided as a Source Data file.

Recent phosphoproteomics analysis of LRRK2 kinase function has identified a subset of Rab GTPases as substrates of LRRK2 kinase activity^31^. In particular, Rab10 and Rab8 were identified as LRRK2 substrates with a potential role in endolysosomal function and lysosomal homeostasis^32^. For these reasons, we sought to examine whether Rab8 or Rab10 were responsible for the regulation of GCase activity by LRRK2. To this end, lentivirus encoding shRNA targeted to Rab10 and Rab8 were generated. Transduction of Rab10 and Rab8 shRNA in fibroblasts led to a significant reduction in the levels of the respective protein relative to scrambled controls (Fig. 5a). Interestingly, knock down of Rab10, but not Rab8, significantly reduced lysosomal GCase activity in the fibroblasts derived from healthy controls and *LRRK2* G2019S PD donors (Fig. 5b–e). Consistent with the greater kinase activity of LRRK2 G2019S, the basal steady state levels of phospho-Rab10 were ~50% higher in G2019S cells as compared to WT controls (Fig. 5f). Similarly, there was ~40% increase in Rab10 phosphorylation in DA neurons derived from *LRRK2* G2019S or R1441C PD donors compared to their respective isogenic controls (Fig. 5h). Treatment of the fibroblasts with MLi-2 significantly (~60%) reduced the levels of phosphorylated Rab10, consistent with previous reports in a variety of cell types (Fig. 5g). However, we did not observe a reduction in GCase protein, indicating that the regulation of GCase activity by LRRK2 is independent of GCase protein expression. Treatment of DA neurons with MLi-2 also significantly (80%) reduced the levels of phosphorylated Rab10 (Fig. 5i).

**Fig. 6:**
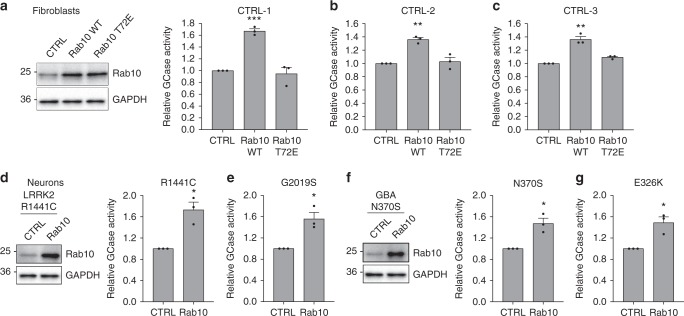
Overexpression of Rab10 WT increases GCase activity in fibroblasts and DA neurons. Relative GCase activity in control fibroblasts transduced with lentivirus expressing GFP, Rab10 WT or Rab10 T72E **a**–**c**. Representative western blots showing overexpression of Rab10 and Rab10 T72E in control (CTRL) fibroblasts (**a** right). Relative GCase activity in DA neurons containing LRRK2 R1441C **d**, LRRK2 G2019S **e**, GBA1 N370S **f**, or GBA1 E326K **g** mutations transduced with CTRL or Rab10 lentivirus for 10 days, with representative western blots showing overexpression of Rab10 in DA neurons (**f** right). The data are presented as the mean ± SEM, *n* = 3; *p < 0.05, **p < 0.01, ***p < 0.001, using one-way ANOVA followed by Tukey’s multiple comparison *post hoc test* **a**–**c**, or paired two-tailed *t*-test **d**–**g**. Source data are provided as a Source Data file.

To further demonstrate a role of Rab10 in regulation of GCase activity, we used lentiviral transduction to express Rab10 WT or phospho-mimic T72E Rab10 in control fibroblasts and examined GCase activity (Fig. 6a). Overexpression of Rab10 WT, but not the phospho-mimic, increased GCase activity in the fibroblasts (Fig. 6a–c). Similarly, overexpression of WT Rab10 in DA neurons with LRRK2 R1441C or G2019S mutations (Fig. 6d, e), as well as neurons with GBA1 N370S or E326K mutations (Fig. 6f, g) resulted in significantly increased GCase activity relative to transduced controls. To confirm that the LRRK2 inhibitors are working directly through LRRK2, we first examined phosphorylation of serine 935, which can be used as a proxy measure of LRRK2 kinase activity^33^. We found that LRRK2 and GBA1 mutant neurons treated with MLi-2 display a reduction in phospho-S935 (Supplementary Fig. 3c, d). Additionally, we employed lentivirus encoding shRNA targeted to LRRK2 to knock down LRRK2 expression in three control fibroblast lines (Supplementary Fig. 3e) and treated these cells with MLi-2 along with the WT cells transduced with scrambled shRNA controls. Consistent with our previous result (Fig. 4f–h), treatment of WT cells with MLi-2 significantly increased GCase activity, however, treatment of LRRK2 knockdown cells with MLi-2 had no effect on GCase activity (Supplementary Fig. 3g–i). Despite the changes in GCase enzymatic activity, we did not observe a change in GCase protein level as a result of LRRK2 or Rab10 knockdown in these cells (Supplementary Fig. 3c). Surprisingly, knockdown of LRRK2 did not have the same effect on GCase activity as treatment with MLi-2 (Supplementary Fig. 3g-i). Further examination of LRRK2 knock-down cells revealed a significant reduction (> 60%) in Rab10 protein levels relative to control transduced cells (Supplementary Fig. 3f). Loss of Rab10 may explain why GCase activity is not increased as a result of LRRK2 knockdown similar to MLi-2 treatment (Supplementary Fig. 3g-i). Together, these data highlight Rab10 as a key mediator of LRRK2 regulation of GCase activity in human fibroblasts and DA neurons.

## Discussion

The recent identification of patients with both * LRRK2* and *GBA1* mutations who exhibit PD symptoms at an earlier age compared to carriers of single *LRRK2* and *GBA1* mutations^22–24^ led us to examine possible mechanistic convergences of *LRRK2* and *GBA1* in PD pathogenesis. In this study, we have shown that GCase activity is reduced in human dopaminergic neurons as a result of LRRK2 mutations. On the other hand, LRRK2 kinase inhibitors restored GCase activity in neurons that carry mutations in *LRRK2* or *GBA1*, and rescued neurons from PD-associated phenotypes including accumulation of dopamine oxidization products and accumulation of phospho-S129 alpha synuclein. Lastly, we have identified the LRRK2 substrate, Rab10, as the key mediator of LRRK2-mediated regulation of GCase activity.

Mutations in *GBA1*, which lead to a reduction in GCase activity, represent an important risk factor for the development of PD^34^. Moreover, we have shown previously that accumulation of misfolded alpha synuclein can also lead to reduction of wild-type GCase in the lysosome^26^, suggesting that the wild-type enzyme activity may be decreased in sporadic forms of PD. This notion was supported by recent studies that found a modest reduction in GCase activity in idiopathic PD patients without *GBA1* mutation^35,36^ highlighting the importance of wild-type GCase activity in idiopathic PD. In addition, we have previously shown that increased levels of cytosolic oxidative stress can promote oxidation of dopamine which can covalently modify GCase at critical catalytic cysteine residues leading to reduced enzymatic activity^19^. This effect was observed in human DA but not mouse DA neurons highlighting physiological differences in handling of dopamine by human neurons which uniquely accumulate neuromelanin^37^. Since an increase in dopamine oxidation was also seen in LRRK2 mutant neurons, we hypothesized that oxidized DA could contribute to the reduction in GCase activity observed in these neurons^12,37^. In LRRK2 mutant neurons, we observe an accumulation of misfolded phospho-S129 alpha synuclein indicating that alpha synuclein accumulation, together with oxidized dopamine,  may also play a potential role in reduced GCase activity in mutant DA neurons.

While these mechanisms may explain the reduction in GCase activity observed in  LRRK2 mutant neurons, we also observed a modest reduction in lysosomal GCase activity in patient-derived fibroblasts with *LRRK2* mutations. Since fibroblasts do not produce dopamine and express only a low level of alpha synuclein, we hypothesized that an alternative mechanism must be responsible for reduction of GCase in these cells. To this end, we identified a direct role of LRRK2 on regulation of GCase activity through Rab10 phosphorylation. This was confirmed by both LRRK2 and Rab10 knock-down, which abolished the effect of treatment with LRRK2 inhibitor on GCase activity in these cells. Interestingly, while live-cell lysosomal GCase activity was significantly increased upon treatment with LRRK2 kinase inhibitor and reduced upon knock-down of Rab10, we observed no change in total cellular GCase protein. This suggests that LRRK2 may regulate the lysosomal environment through Rab10 which is consistent with the recent observation of the role of Rab10 in lysosomal homeostasis^32^.

In our experiments, increased Rab10 phosphorylation was comparable to Rab10 knock down in reducing GCase activity, consistent with the recent report that phosphorylation of Rab10 by LRRK2 leads to deactivation of Rab10 protein function^11^. Interestingly, while inhibition of LRRK2 kinase activity significantly increased GCase activity in patient-derived fibroblasts, knock-down of LRRK2 had an opposite effect and reduced GCase activity. This apparently contradictory result could potentially be explained by the significant decrease in Rab10 levels seen in these cells as a result of LRRK2 knock-down that is not observed upon kinase inhibition. Exactly how LRRK2 knock-down effects Rab10 protein levels remains of considerable interest and requires additional investigation. The current data suggest that some additional function of LRRK2, independent of LRRK2 kinase activity, is affected by knock-down of the protein. This could be through loss of LRRK2 GTPase activity^38^ or loss of LRRK2 scaffolding function in regulation of signaling cascades^39^.

Considerable efforts have been focused on development of therapeutic agents to target LRRK2 and GCase. LRRK2 kinase inhibitors are currently in phase 1b clinical trials in PD patients with and without *LRRK2* mutations (NCT03710707). Our results define a mechanism through which LRRK2 inhibition could be therapeutically beneficial for the treatment of the broader PD population, as well as for *GBA1*-PD patients. In addition, recent observations have described an increase in WT LRRK2 kinase activity in idiopathic PD^11^. Intriguingly, based on our results, this increase in LRRK2 activity in idiopathic PD may also contribute to reduced GCase activity in the absence of *GBA1* mutations^35,36^. In addition to LRRK2 inhibition, activation of GCase is another strategy that has exciting therapeutic potential^34^. GCase activators are also currently in clinical trials for individuals with *GBA1*-PD (NL7061, NL6574). While these trials are currently limited to patients with *GBA1*-PD, our observation that GCase is consistently reduced in LRRK2 mutant neurons suggest that *LRRK2* patients could benefit from GCase activation. In conclusion, our results highlight the potential for existing therapeutic strategies targeting LRRK2 and GCase to have a broader application for PD patients.

## Methods

### Antibodies

For western blot analysis rabbit anti-rab10 (Cell Signaling, 8127S, 1:1000), rabbit anti-phospho-rab10 (Abcam, ab230261, 1:1000), rabbit anti-rab8 (Cell signaling, 6971S, 1:1000), mouse anti-β-3-tubulin (Cell signaling, 4466S, 1:4000), rabbit anti-TH (Millipore, 657012, 1:4000), rabbit anti pS129 aSyn (Abcam, ab51253, 1:1000), mouse anti alpha synuclein (BD Biosciences, 610787, 1:2000), rabbit anti-LRRK2 (Abcam, ab133474, 1:500), rabbit anti-phospho S935 LRRK2 (Abcam, ab133450, 1:500). rabbit anti-alpha synuclein (Santacruz, SC7011-R, 1:2000), rabbit anti-Glucocerebrosidase (Sigma, G4171), mouse anti-alpha tubulin (Sigma, T5168, 1:40,000), mouse anti-GAPDH (Millipore, MAB374, 1:5000). The secondary antibodies used in western blot analysis goat anti-mouse and goat anti-rabbit (Jackson ImmunoResearch lab, #115–035–146, #111–035–144, 1:10,000). For immunocytochemistry, rabbit anti-pS129 aSyn (Abcam, ab51253, 1:200), mouse anti-β-3-tubulin (BioLegend, 801202, 1:500), rabbit anti-β-3-tubulin (BioLegend, 802001, 1:500), sheep anti-TH (Novus, NB300–110, 1:500) mouse anti-MAP2 (Sigma, M4403, 1:500).

### Human iPSC culture and differentiation

All cell lines were obtained from the Northwestern University Biorepository or from the NINDS Human Cell and Data Repository. To ensure that the cell lines did not contain unknown *GBA1* mutations, all exons of *GBA1* were sequenced in the cell lines examined. Additionally, exon 31 and 41 of *LRRK2*, which contain the overwhelming majority of *LRRK2* mutations were also sequenced. Human iPSCs were generated from skin fibroblasts through retroviral expression of OCT4, SOX2, cMyc, KLF4^40^. iPSCs were validated by qPCR analysis comparing expression of classical stem cell markers relative to embryonic stem cells including KLF4, UTF1, DNMT3B, SOX2, cMyc, OCT4, Nanog, and TDGF1 (Supplementary Fig. 1a-d) using established primers (Supplementary Methods). For qPCR, RNA was isolated using RNeasy kit (Qiagen) and transcribed to cDNA using the High-Capacity cDNA Reverse Transcription Kit (ThermoFisher). qPCR was performed in the 7500 Fast Real-Time PCR system (Applied Biosystems), using ssoAdvanced universal SYBR Green Supermix (BioRad). All lines were routinely tested for mycoplasma contamination using PCR based detection (Venor GeM Mycoplasma Detection Kit (Sigma, MP0025)).

iPSCs were continuously maintained in mTeSR1 (StemCell Technologies) with manual passaging once a week prior to differentiation. Dopaminergic neuron differentiation was performed using the Kriks et al. protocol^25^. Cells were passaged in 1 mm chunks on day 13 to 10 cm dishes coated with poly-D-lysine (PDL)/laminin. At day 25, cells were treated with Accutase to achieve single cell populations and plated onto PDL/laminin coated tissue culture plates. Following this final passage, cells were fed every 3 days with Neurobasal Media (Gibco) + SM1 supplement (Stemcell Technologies) containing BDNF, ascorbic acid, GDNF, TGF-β3, cAMP, and DAPT until day 40. After day 40, cells were maintained in Neurobasal + SM1 supplement twice weekly. Quality control was performed by immunocytochemistry on each differentiation set to ensure efficient differentiation (β-III-tubulin positive > 99%) with equivalent expression of midbrain specific markers among compared lines (TH > 60%) (Supplementary Fig. 2)^19^.

### Generation of isogenic lines

Optimal CRISPR guides were chosen using the CRISPOR design tool^41^ to target *LRRK2* or *GBA1* mutation. Guides were cloned into pSpCas9(BB)-2A-GFP (pX458, Addgene #48138) and Sanger sequenced to ensure proper cloning. iPSC colonies grown on a 10 cm dish were dissociated using Accutase and 5 million cells were transduced with 3 µg CRISPR guide, along with 4 µg ssODN using the Neon transfection system (Thermo Fisher). Transduced cells were then plated using mTeSR with 10 µM ROCK inhibitor. After 48 h, GFP positive cells were sorted and plated at clonal density (10,000 cells/plate) on Matrigel coated 10 cm dishes. Individual colonies were manually passaged and plated in 48 well plates. Clones were grown to confluence and passaged using Accutase. About 15% of cells were replated and the remaining were used for Sanger sequencing reactions. Crude genomic DNA was obtained using Viagen extraction reagents. Corrected clones were expanded, resequenced, and submitted for g-band karyotype analysis (Cell Line Genetics).

### Treatment conditions

Differentiated dopaminergic neurons 80 days in culture were treated every 2 days for 12 days with 600 nM MLi-2 which significantly inhibits LRRK2, or 3 µM 6166 which was found previously to promote robust GCase modulation^29^ prior to analysis. Treatments for GCase activity measurements and biochemical assays were performed simultaneously on neurons derived from identical differentiations. Sample numbers listed in the figure legend represent separate DA neuron differentiations. For assays using fibroblasts, cells were pre-treated for 3 days with 600 nM MLi-2, 2 µM LRRK2-IN-1^42^ and 3 µM 6166^29^ prior to analysis.

### Live cell GCase assay

At day 25 after differentiation, dopaminergic neural precursor cells were dissociated and plated on black 96-well plates (40,000 cells/well) coated with poly-D-lysine and laminin. GCase activity was assessed in culture using the fluorescent quenched substrate 5-(pentafluorobenzoylamino) fluorescein di-β-D-glucopyranoside (PFB-FDGlu) (Thermo Fisher). Following a wash with phenol red free Neurobasal media, 100 µL of phenol red free Neurobasal + SM1 supplement containing 50 µg/mL PFB-FDGlu was added to each well. Supplemental wells were also treated with 400 nM bafilomycin A1. Cells were preincubated for 60 min at 37 °C to allow for the substrate to accumulate in the lysosome and hydrolysis was monitored using a plate reader to monitor unquenching of the fluorescent probe every 15 min for 90 min at 485/525 nm (excitation/emission). For fibroblasts, substrate was pre-incubated for 75 min and plates were read every 20 min for 120 min. Lysosomal hydrolysis of the substrate was determined by subtracting the signal obtained from bafilomycin A1 treated cells from the total fluorescence intensity. Lysosomal GCase activity was considered the bafilomycin A1 sensitive fraction of the fluorescent signal. Following the activity measurements, cells were washed in 1× PBS, lysed in RIPA buffer, and the total protein concentration was determined using the standard BCA Assay. Lysosomal GCase activity was then normalized to total protein concentration.

### Western blot analysis

To obtain samples for western blot analysis, media was aspirated from neurons plated on 12-well or 6-well plates. Cells were washed in 1× PBS, scraped off the plate, pelleted in 1.5 mL microcentrifuge tubes, and stored at −80 °C until further use. The cells were lysed on ice in RIPA buffer (50 mM Tris HCL, pH 7.4, 150 mM NaCl, 0.1% (w/v) SDS, 0.5 % (w/v) sodium deoxycholate, 1% triton X-100) with Halt^TM^ protease/phosphatase inhibitor cocktail (Thermo Scientific). Lysates were cleared by centrifugation at 20,000 × *g*, and the protein concentration was determined using a BCA protein assay kit. Lysates were denatured by heating in 4X Laemmli sample buffer (BioRad). Equal amounts of protein were separated using precast, 4–12% or 4–20% Tris-glycine gels. Proteins were transferred onto PVDF membrane using the Transblot Turbo transfer system (BioRad). Blots used to detect alpha-synuclein or Rab proteins were pre-fixed in 0.5% paraformaldehyde for 20 min. Membranes were blocked with 10% milk in TBS-T (50 mM tris, pH 7.4, 150 mM NaCl, 0.1% Tween20). Membranes were incubated with primary antibody overnight at 4 °C. After washing, membranes were incubated with horseradish peroxidase-conjugated anti-rabbit or anti-mouse secondary antibody for 1.5 h at room temperature. Chemiluminescence was assessed using pico or femto chemiluminescence substrates (Thermo Fisher). Bands were imaged on a ChemiDoc XRS + imaging station with a 16-bit CCD camera. Quantification of protein bands was done using ImageJ software (NIH). Normalization was performed using β-3-tubulin signal in neurons and α-tubulin or GAPDH signal in fibroblasts.

### Near infrared fluorescence detection of oxidized dopamine

Accumulation of neuromelanin-like dopamine oxidation products was measured, as previously described^19^ with some modification. Briefly, dopaminergic neurons were lysed with RIPA buffer and centrifuged at 20,000 × *g* for 20 min. The protein concentration from the resulting supernatant was determined using a BCA Assay. Protein in the resulting insoluble material was extracted by resuspending the pellet in 1× PBS with 2% SDS, boiled for 5 min, sonicated, and boiled for an additional 5 min. The samples were then centrifuged at 100,000 × *g* for 20 min and the supernatant was removed. The resulting pellet was extracted in 4 N NaOH overnight at 55 °C. The total protein in the lysate was used to determine the volume of 4 N NaOH to add to the insoluble pellet to compensate for differences in starting cell number, ensuring equal loading. In total, 10 µl was blotted onto Biodyne B nylon membrane (Pall Life Sciences). Membranes were imaged on the Odyssey CLx (LI-COR Biosciences) using the 700 nm excitation wavelength. Spot intensity was analyzed using ImageJ software (NIH).

### Immunocytochemistry

Neurons plated on nitric acid-treated coverslips were washed with 1× PBS and fixed using 4% paraformaldehyde for 10 min at room temperature. Cells were permeabilized using 0.1% saponin in 1× PBS, then blocked in 1× PBS, 0.1% saponin, 10% FBS, 1% BSA for 20 min. Fixed cells were incubated with primary antibodies overnight at 4 °C, washed three times in 1× PBS, then incubated with secondary antibodies for 1 h at room temperature. Coverslips were mounted onto slides using Vectashield + DAPI (Vector Labs). Images were acquired on the Leica DMI4000B or the Nikon A1R confocal microscopes.

### Generation of lentiviral constructs

Rab8, Rab10, and LRRK2 shRNA constructs were obtained from Sigma. Rab10 WT and T72E were cloned into the pER4 lentiviral expression vector using standard cloning techniques. Lentiviral vectors along with helper plasmids, pLP3 (Invitrogen) and psPAX2 (Addgene, #12260), were transfected into HEK-FT cells using X-treme Gene HP DNA Transfection Reagent (Roche). The resulting virus particles were purified and concentrated using LentiX-concentrator (Clontech). Virus titers were determined using the RETROtek p24 Antigen ELISA kit (ZeptoMetric Corporation). Concentrated virus was stored in single use aliquots at −80 °C.

### Statistical analysis

Statistical calculations were performed using GraphPad Prism 7 software. Datasets with two samples were analyzed using a two-tailed *t*-test. Datasets with more than two samples were analyzed using one-way ANOVA followed by Tukey’s post-hoc test.

### Reporting summary

Further information on research design is available in the Nature Research Reporting Summary linked to this article.

## Supplementary information

Supplementary Information

Reporting Summary

## Data Availability

Sequence data that support the findings of this study have been deposited at https://zenodo.org/record/3531884#.XcQ7WlX7SUk. All other data that support the findings of this study are included in Source Data file accompanying the manuscript or are available from the authors upon reasonable request.

## Source data

Source Data
