# Supplementary material for: LRRK2 kinase activity regulates lysosomal glucocerebrosidase in neurons derived from Parkinson’s disease patients
Source: Nat Commun. 2019 Dec 5;10:5570. doi: 10.1038/s41467-019-13413-w (PMC6895201; doi:10.1038/s41467-019-13413-w)
Supplement: Supplementary file 1 — Supplementary Information [file 41467_2019_13413_MOESM1_ESM.pdf]

Supplemental Information

LRRK2 kinase activity regulates lysosomal glucocerebrosidase in neurons derived from Parkinson's disease patients

Daniel Ysselstein[1], Maria Nguyen[1], Tiffany Young[1], Alex Severino[1], Michael Schwake[1], Kalpana Merchant[1], Dimitri Krainc[1*]

[1]Department of Neurology, Northwestern University Feinberg School of Medicine, 303 E Chicago Avenue, Chicago, Illinois 60611, USA

[*]To whom correspondence should be addressed: Email: dkrainc@nm.org

Supplementary Methods
QPCR primers used in this study
KLF4
Forward: AGTCTCCAAGCGACGAAAAA
Reverse: GACCTGGAAAATGCTCGGTC

UTF-1
Forward: CCGTCGCTGAACACCGCCCTGCTG
Reverse: CGCGCTGCCCAGAATGAAGCCCAC

DNMT3B
Forward: GCTCACAGGGCCCGATACTT
Reverse: GCAGTCCTGCAGCTCGAGTTTA

SOX2
Forward: TGGCGAACCATCTCTGTGGT
Reverse: CCAACGGTGTCAACCTGCAT

OCT4
Forward: GGAAGGAATTGGGAACACAAAGG
Reverse: AACTTCACCTTCCCTCCAACCA

cMyc
Forward: CCAGCAGCGACTCTGAGGA
Reverse: GAGCCTGCCTCTTTTCCACAG

Nanog
Forward: CCTGTATTTGTGGGCCTG
Reverse: GACAGTCTCCGTGTGAGGCAT

TDGF1
Forward: CTCCTGCCTGAATGGGGGAACCTGC
Reverse: GCCACGAGGTGCTCATCCATCACAAGG

B-Actin
Forward: CTGTCCCTGTATGCCTCTG
Reverse: ATGTCACGCACGATTTCC

Supplementary Figures

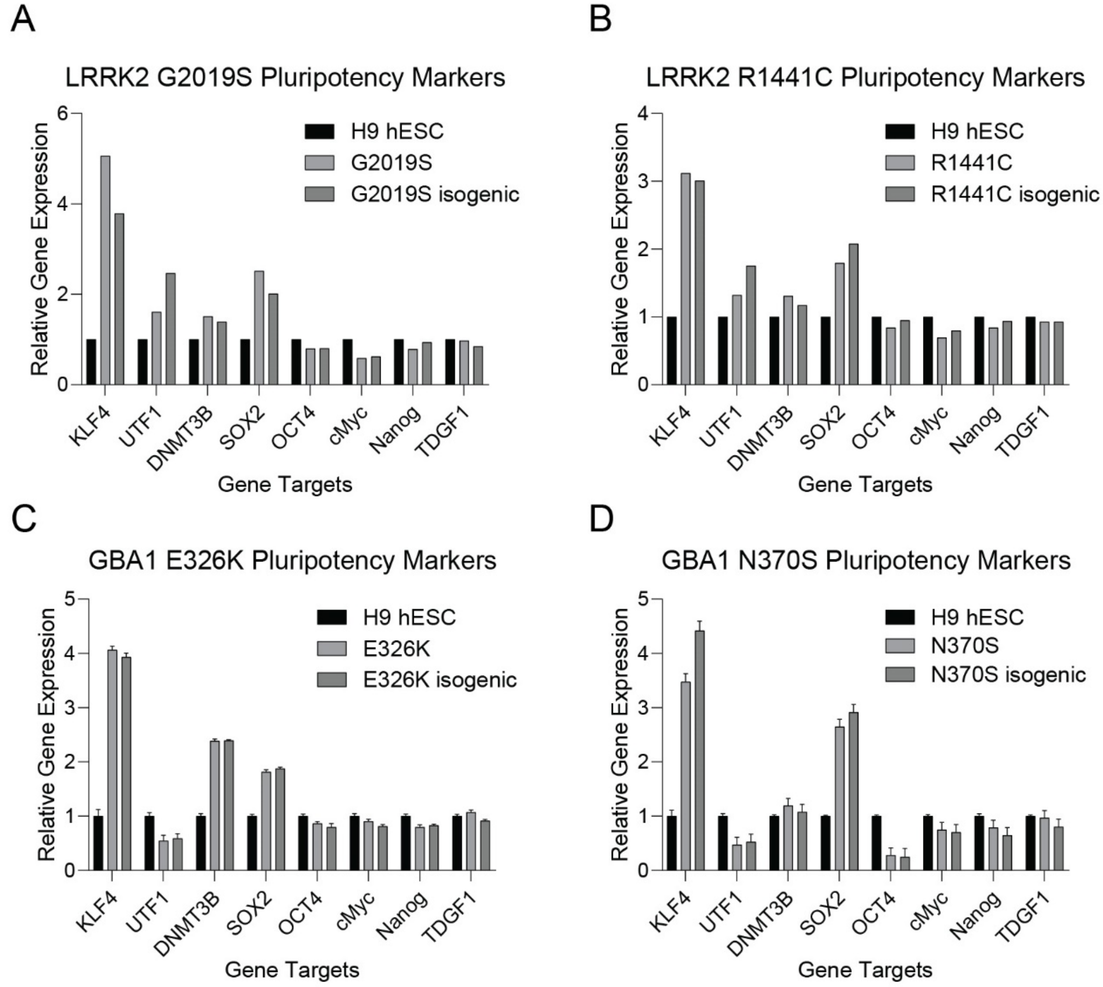

Supplementary Figure 1. Characterization of isogenic corrected LRRK2 iPSC lines. qRT-PCR analysis for the expression of pluripotency markers KLF4, UTF1, DNMT3B, SOX2, OCT4, cMyc, Nanog, and TDGF1 normalized to β-actin, used as a housekeeping gene, in LRRK2 G2019S (**A**), R1441C (**B**), *GBA1* N370S (**C**), and E326K (**D**) mutant iPSCs along with isogenic controls. Expression is presented as fold change relative to H9 human embryonic stems cells. Source data are provided as a Source Data file.

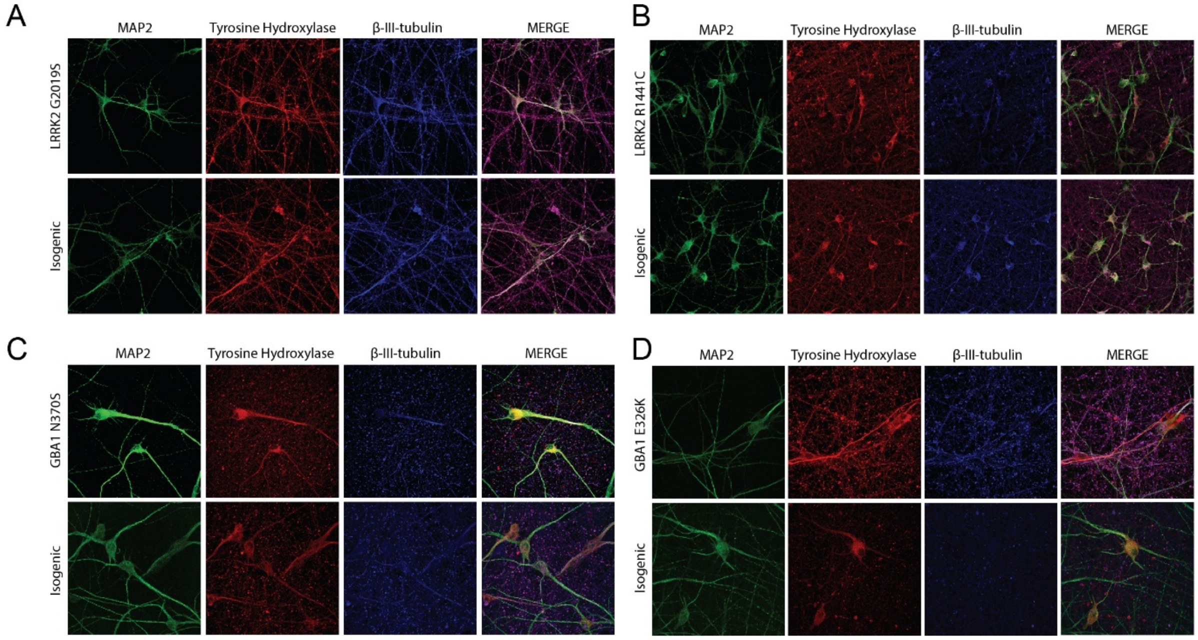

Supplementary Figure 2. Characterization of LRRK2 DA neurons. Immunocytochemistry of representative LRRK2 G2019S (**A**), R1441C (**B**), *GBA1* N370S (**C**), and E326K (**D**) DA neurons along with corresponding isogenic controls using antibodies against MAP2, Tyrosine Hydroxylase, and β-III-tubulin.

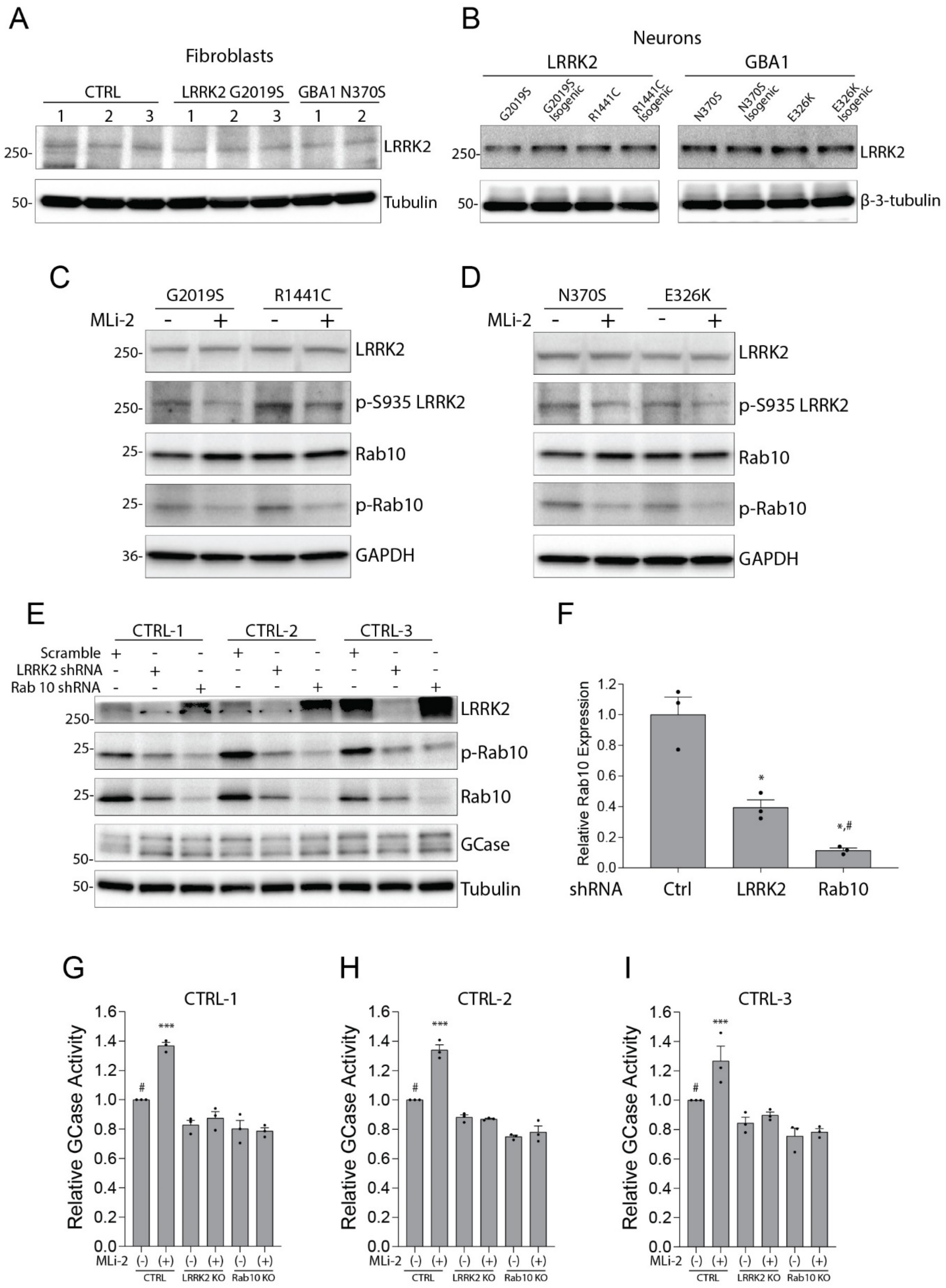

A
Fibroblasts
CTRL
LRRK2 G2019S
GBA1 N370S
1 2 3 1 2 3 1 2
250-
LRRK2
50-
Tubulin
B
Neurons
LRRK2
GBA1
G2019S
G2019S Isogenic
R1441C
R1441C Isogenic
N370S
N370S Isogenic
E326K
E326K Isogenic
250-
LRRK2
50-
β-3-tubulin
C
G2019S
R1441C
MLi-2 - + - +
250-
LRRK2
250-
p-S935 LRRK2
25-
Rab10
25-
p-Rab10
36-
GAPDH
D
N370S
E326K
MLi-2 - + - +
LRRK2
p-S935 LRRK2
Rab10
p-Rab10
GAPDH
E
CTRL-1
CTRL-2
CTRL-3
Scramble
LRRK2 shRNA
Rab 10 shRNA
250-
LRRK2
25-
p-Rab10
25-
Rab10
50-
GCase
50-
Tubulin
F
Relative Rab10 Expression
shRNA
Ctrl
LRRK2
Rab10
*
*,#
G
CTRL-1
H
CTRL-2
I
CTRL-3
Relative GCase Activity
MLi-2
(-) (+) (-) (+) (-) (+)
CTRL
LRRK2 KO
Rab10 KO
#
***

Supplementary Figure 3. Specificity of LRRK2 and Rab10 in MLi-2 mediated changes in GCase activity. Western blot analysis of lysates from fibroblasts (**A**) and neurons (**B**) cell lines used in this study probed for LRRK2 with tubulin and β-3-tubulin as a loading control. Western blot analysis of lysates from neurons with LRRK2 G2019S or R1441C mutation (**C**) and *GBA1* N370S or E326K mutation (**D**) treated with MLi-2 probed for LRRK2, phospho-S935 LRRK2, phospho-Rab10, Rab10, and tubulin (loading control). (**E**) Western blot analysis of lysates from 3 control fibroblast lines transduced with lentivirus encoding LRRK2 and Rab10 or a scrambled control probed for LRRK2, phospho-Rab10, Rab10, GCase, and tubulin (loading control). Relative expression of Rab10 is quantified (**F**). Examination of lysosomal GCase activity in CTRL, LRRK2 and Rab10 knockdown fibroblasts treated for 96 h with MLi-2 (**D-F**). The data are presented as the mean ± SEM, n=3, (**F**) *p<0.01 LRRK2 and Rab10 KD relative to CTRL, #p<0.05 for Rab10 KD relative to LRRK2 KD, (**G-I**) ***p<0.001 for MLi-2 treated CTRL relative to all other conditions, (**G-I**) #p<0.05 for CTRL relative to Rab10 KD, (**G,H**) #p<0.05 for CTRL relative to LRRK2 KO, using one-way ANOVA followed by Tukey's multiple comparison *post hoc test*. Source data are provided as a Source Data file.
