# Supplementary material for: LRRK2 kinase activity regulates lysosomal glucocerebrosidase in neurons derived from Parkinson’s disease patients
Source: Nat Commun. 2019 Dec 5;10:5570. doi: 10.1038/s41467-019-13413-w (PMC6895201; doi:10.1038/s41467-019-13413-w)
Supplement: Supplementary file 2 — Reporting Summary [file 41467_2019_13413_MOESM2_ESM.pdf]

nature research

Corresponding author(s): Dimitri Krainc

Last updated by author(s): Oct 25, 2019

Reporting Summary

Nature Research wishes to improve the reproducibility of the work that we publish. This form provides structure for consistency and transparency in reporting. For further information on Nature Research policies, see Authors & Referees and the Editorial Policy Checklist.

Statistics

For all statistical analyses, confirm that the following items are present in the figure legend, table legend, main text, or Methods section.

n/a	Confirmed	
☐	☒	The exact sample size (*n*) for each experimental group/condition, given as a discrete number and unit of measurement
☐	☒	A statement on whether measurements were taken from distinct samples or whether the same sample was measured repeatedly
☐	☒	The statistical test(s) used AND whether they are one- or two-sided<br>*Only common tests should be described solely by name; describe more complex techniques in the Methods section.*
☐	☒	A description of all covariates tested
☐	☒	A description of any assumptions or corrections, such as tests of normality and adjustment for multiple comparisons
☐	☒	A full description of the statistical parameters including central tendency (e.g. means) or other basic estimates (e.g. regression coefficient) AND variation (e.g. standard deviation) or associated estimates of uncertainty (e.g. confidence intervals)
☒	☐	For null hypothesis testing, the test statistic (e.g. *F*, *t*, *r*) with confidence intervals, effect sizes, degrees of freedom and *P* value noted<br>*Give P values as exact values whenever suitable.*
☒	☐	For Bayesian analysis, information on the choice of priors and Markov chain Monte Carlo settings
☒	☐	For hierarchical and complex designs, identification of the appropriate level for tests and full reporting of outcomes
☒	☐	Estimates of effect sizes (e.g. Cohen's *d*, Pearson's *r*), indicating how they were calculated

Our web collection on statistics for biologists contains articles on many of the points above.

Software and code

Policy information about availability of computer code

Data collection	*Provide a description of all commercial, open source and custom code used to collect the data in this study, specifying the version used OR state that no software was used.*
Data analysis	GraphPad Prism (v7.0) was used for statistical analysis and data visualization. ImageJ (NIH) was used for densitometric quantification of western blots.

For manuscripts utilizing custom algorithms or software that are central to the research but not yet described in published literature, software must be made available to editors/reviewers. We strongly encourage code deposition in a community repository (e.g. GitHub). See the Nature Research guidelines for submitting code & software for further information.

Data

Policy information about availability of data

All manuscripts must include a data availability statement. This statement should provide the following information, where applicable:

- Accession codes, unique identifiers, or web links for publicly available datasets
- A list of figures that have associated raw data
- A description of any restrictions on data availability

Sequence data that support the findings of this study have been deposited in the Mendeley Data repository (DOI: 10.17632/r49wwrw9st.3). All other data that support the findings of this study are included in Source Data file accompanying the manuscript or are available from the authors upon reasonable request.

Field-specific reporting

Please select the one below that is the best fit for your research. If you are not sure, read the appropriate sections before making your selection.

☒ Life sciences ☐ Behavioural & social sciences ☐ Ecological, evolutionary & environmental sciences

For a reference copy of the document with all sections, see nature.com/documents/nr-reporting-summary-flat.pdf

Life sciences study design

All studies must disclose on these points even when the disclosure is negative.

Sample size	No sample size calculation was used to predetermine sample size. Sample size was chosen to achieve statistical significance and minimize cost
Data exclusions	No data was excluded from the analysis.
Replication	All experiments were performed on neurons generated in separate differentiations.
Randomization	*Describe how samples/organisms/participants were allocated into experimental groups. If allocation was not random, describe how covariates were controlled OR if this is not relevant to your study, explain why.*
Blinding	Blinding was not performed

Reporting for specific materials, systems and methods

We require information from authors about some types of materials, experimental systems and methods used in many studies. Here, indicate whether each material, system or method listed is relevant to your study. If you are not sure if a list item applies to your research, read the appropriate section before selecting a response.

Materials & experimental systems

n/a	Involved in the study	
☐	☒	Antibodies
☐	☒	Eukaryotic cell lines
☒	☐	Palaeontology
☒	☐	Animals and other organisms
☒	☐	Human research participants
☒	☐	Clinical data

Methods

n/a	Involved in the study	
☒	☐	ChIP-seq
☒	☐	Flow cytometry
☒	☐	MRI-based neuroimaging

Antibodies

Antibodies used	For western blot analysis rabbit anti-rab10 (Cell Signaling, 8127S, 1:1000), rabbit anti-phospho-rab10 (Abcam, ab230261, 1:1000), rabbit anti-rab8 (Cell signaling, 6971S, 1:1000), mouse anti-β-3-tubulin (Cell signaling, 4466S, 1:4000), rabbit anti-TH (Millipore, 657012, 1:4000), rabbit anti pS129 aSyn (Abcam, ab51253, 1:1000), mouse anti alpha synuclein (BD Biosciences, 610787, 1:2000), rabbit anti-LRRK2 (Abcam, ab133474, 1:500), rabbit anti-phospho S935 LRRK2 (Abcam, ab133450, 1:500). rabbit anti-alpha synuclein (Santacruz, SC7011-R, 1:2000), rabbit anti-Glucocerebrosidase (Sigma, G4171), mouse anti-alpha tubulin (Sigma, T5168, 1:40,000), mouse anti-GAPDH (Millipore, MAB374, 1:5000). The secondary antibodies used in western blot analysis goat anti-mouse and goat anti-rabbit (Jackson ImmunoResearch lab, #115-035-146, #111-035-144, 1:10,000). For immunocytochemistry, rabbit anti-pS129 aSyn (Abcam, ab51253, 1:200), mouse anti-β-3-tubulin (BioLegend, 801202, 1:500), rabbit anti-β-3-tubulin (BioLegend, 802001, 1:500), sheep anti-TH (Novus, NB300-110, 1:500) mouse anti-MAP2 (Sigma, M4403, 1:500).
Validation	In addition to claims made by manufacturers, prior to use, new antibodies were routinely validated.

Eukaryotic cell lines

Policy information about cell lines

Cell line source(s)	Primary patient fibroblast with LRRK2 mutations were obtained from NINDS Human Cell and Data Reository. All other primary patient fibroblast were obtained from the Northwestern University Biorepostory.
Authentication	All specific point mutations were confirmed by sequence. Additional sequencing was performed to determine the absence of additional common LRRK2 and GBA1 mutations.

Mycoplasma contamination	All cell lines were routinely tested for mycoplasma testing using a PCR based detection (Venor GeM Mycoplasma Detection Kit (Sigma, MP0025
Commonly misidentified lines<br>(See ICLAC register)	None
